# Machine learning-based ultrasound radiomics for predicting risk of recurrence in breast cancer

**DOI:** 10.3389/fonc.2025.1542643

**Published:** 2025-05-12

**Authors:** Wei Fan, Hao Cui, Xiaoxue Liu, Xudong Zhang, Xinran Fang, Junjia Wang, Zihao Qin, Xiuhua Yang, Jiawei Tian, Lei Zhang

**Affiliations:** ^1^ Department of Ultrasound Medicine, the First Affiliated Hospital of Harbin Medical University, Heilongjiang, China; ^2^ Department of Ultrasound Medicine, the Second Affiliated Hospital of Harbin Medical University, Heilongjiang, China

**Keywords:** radiomics, breast cancer, recurrence risk, ultrasound, nomogram

## Abstract

**Purpose:**

To develop a radiomics model based on ultrasound images for predicting risk of recurrence in breast cancer patients.

**Methods:**

In this retrospective study, 420 patients with pathologically confirmed breast cancer were included, randomly divided into training (70%) and test (30%) sets, with an independent external validation cohort of 90 patients. According to St. Gallen recurrence risk criteria, patients were categorized into two groups, low-medium-risk and high-risk. Radiomics features were extracted from a radiomics analysis set using Pyradiomics. The informative radiomics features were screened using the minimum redundancy maximum relevance (mRMR) and the least absolute shrinkage and selection operator (LASSO) algorithms. Subsequently, radiomics models were constructed with eight machine learning algorithms. Three distinct nomogram models were created using the features selected through multivariate logistic regression, including the Clinic-Ultrasound (Clin-US), Clinic-Radiomics (Clin-Rad), and Clinic-Ultrasound-Radiomics (Clin-US-Rad) models. The receiver operating characteristic (ROC), calibration, and decision curve analysis (DCA) curves were used to evaluate the model’s clinical applicability and predictive performance.

**Results:**

A total of 12 ultrasound radiomics features were screened, of which wavelet.LHL first order Mean features weighed more and tended to have a high risk of recurrence. The higher the risk of recurrence, the higher the radiomics score (Rad-score) in all three sets (training, test, and external validation set, all *p* < 0.05). Rad-score is equally applicable in four different subtypes of breast cancer. In the test set and external validation set, the Clin-US-Rad model achieved the highest AUC values (AUC = 0.817 and 0.851, respectively). The calibration and DCA curves also demonstrated the good clinical utility of the combined model.

**Conclusion:**

The machine learning-based ultrasound radiomics model were useful for predicting the risk of recurrence in breast cancer. The nomograms show promising potential in assessing the recurrence risk of breast cancer. This non-invasive approach offers crucial guidance for the diagnosis and treatment of the condition.

## 1 Introduction

Breast cancer is the most common type of cancer worldwide, and it continues to be the primary cause of cancer-related mortality among women (1, 2). Although many standardized diagnostic methods and treatment modalities have been advanced, many patients remain at risk of recurrence and metastasis (3). Current methods for assessing recurrence risk, such as Genetic tests (e.g., Oncotype DX 21-gene Recurrence Score and Mammaprint), are valuable but limited by their invasiveness, high cost, and postoperative nature (4). The St. Gallen International Expert Consensus has gained prominence in treating and managing breast cancer (5–7). According to this criteria, patients with breast cancer can be classified into low-, medium-, and high-risk categories (8). This criterion relies heavily on postoperative pathological findings, such as histological grade, immunohistochemistry, and the status of the axillary lymph nodes. However, there are some critical gaps in preoperative noninvasive risk assessment. Preoperative accurate assessment of recurrence risk is crucial for guiding personalized treatment strategies (9–11). For patients with high-risk need more aggressive interventions, while patients with low-medium risk need avoid overtreatment. Medical imaging provides extensive information regarding the lesion and can be utilized to assess the tumor’s biological behavior comprehensively. Moreover, medical imaging is a noninvasive, cost-effective, and easily accessible method (12). Currently, the absence of image-based tools for recurrence risk prediction limits clinical decision-making. Therefore, there is an urgent need to develop a more convenient and appropriate preoperative noninvasive method for predicting breast cancer recurrence risk.

Ultrasound is a convenient, economical, universally applicable, and real-time dynamic examination method that has become the preferred method for diagnosing breast lesions in China (13, 14). Meanwhile, ultrasound features are strongly associated with the recurrence risk of breast cancer. Breast cancer at high risk of recurrence is associated with growth orientation, margin, posterior acoustic pattern, and breast imaging reporting and data system (BI-RADS) grade. For example, Wang et al. (15) showed that vertically growing triple-negative breast cancer has a short relapse-free survival. Zhang et al. (16) showed that breast cancers with indistinct margins are more aggressive and have a higher risk of recurrence. Luo et al. (17) demonstrated a correlation between posterior echo enhanced and high-risk indicators of breast cancer, which means a high risk of recurrence and a poor prognosis. Presently, there is lack of methods to integrate BI-RADS ultrasound features into models to predict recurrence risk of breast cancer.

Ultrasound radiomics, is an emerging method to extract high-throughput features from medical ultrasound images based on machine learning algorithms (18, 19). Ultrasound radiomics features can already differentiate between benign and malignant breast tumors, predict axillary lymph node metastases, and determine the histological grade of breast cancer (20–22). A relatively small number of studies have been conducted on the application of ultrasound radiomics to predict breast cancer recurrence and metastasis. Hence, it is important to investigate the correlation between ultrasound radiomics features and breast cancer recurrence risk. Additionally, nomograms serve as a visual tool to integrate multiple risk features into a single, easy-to-interpret graphical representation, which enhances their clinical utility and predictive accuracy (23).

Here, we aimed to establish a preoperative, non-invasive radiomics model that combined BI-RADS ultrasound and clinical features to predict the risk of breast cancer recurrence. By integrating these complementary features into an easy-to-interpret nomogram, our model enables personalized risk stratification, facilitating clinically tailored treatment decisions. Furthermore, we evaluated the predictive efficacy of different feature combinations to identify the one most closely associated with clinical practice.

## 2 Methods

**Figure 1:**
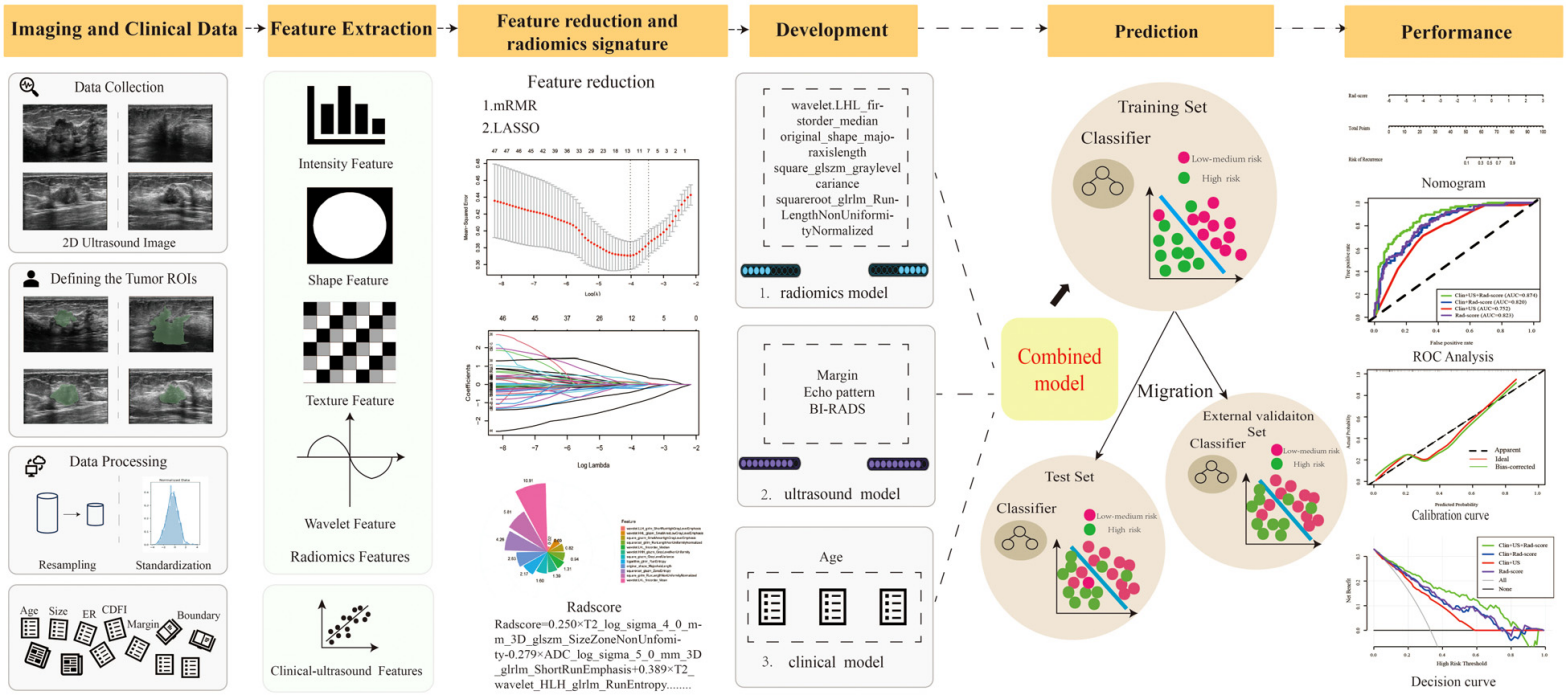
The pipeline of this study. (mRMR, Max-Relevance and Min-Redundancy; LASSO, least absolute shrinkage and selection operator; Rad-score, Radiomics score).

**Figure 1** is the research flowchart. The present study utilized radiomics to predict the risk of recurrence in breast patients in combination with radiomics, BI-RADS ultrasound, and clinical features.

### 2.1 Patients

**Figure 2:**
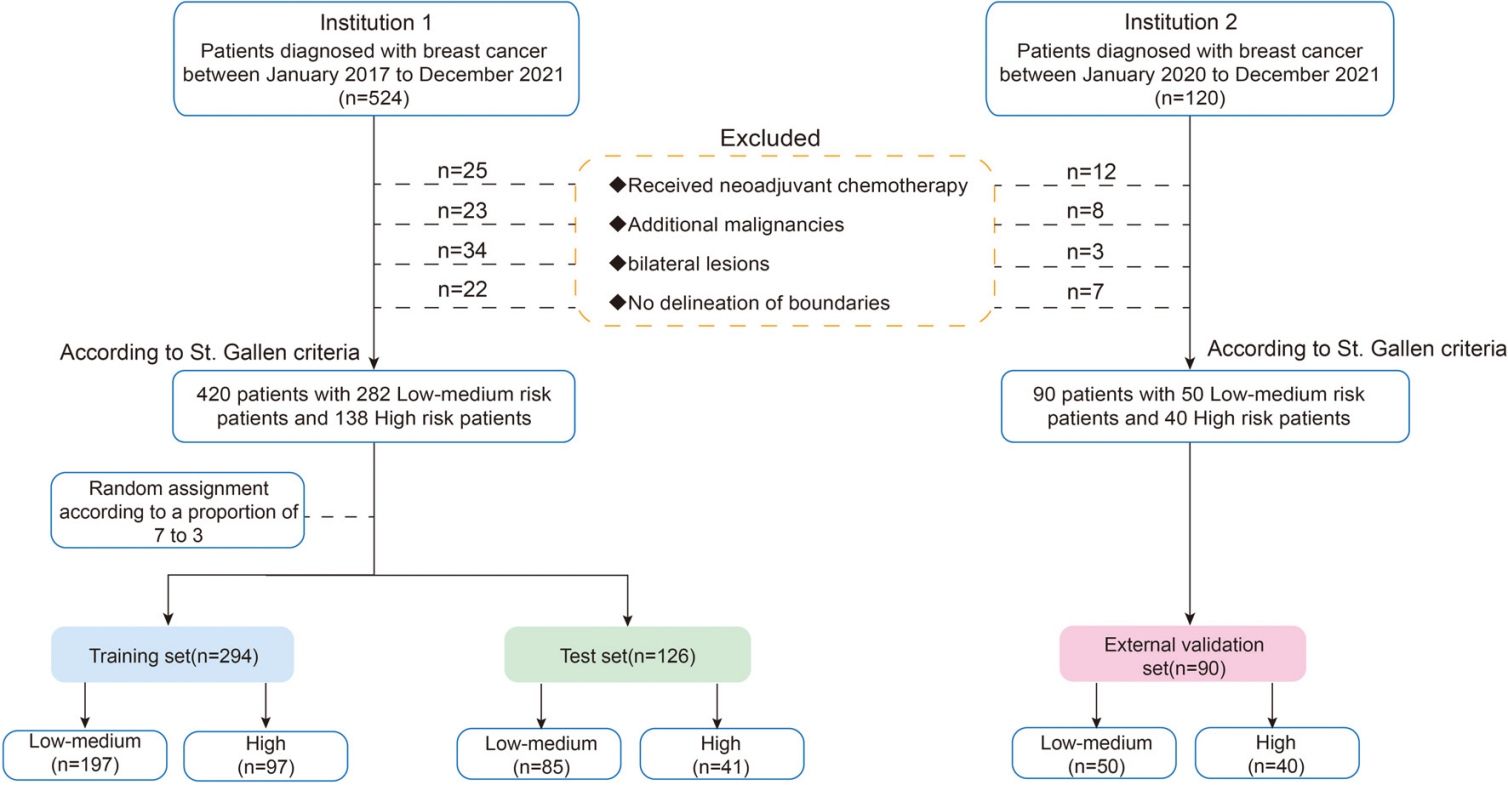
Flow chart of patient enrolment.

Patients were continuously recruited from the Second Affiliated Hospital of Harbin Medical University (Institution 1) from January 2017 to December 2021. The inclusion criteria were as follows: (1) pathologically confirmed invasive breast cancer; (2) preoperative ultrasound examination with complete ultrasound images; (3) available postoperative pathology and immunohistochemistry information. The exclusion criteria included the following: (1) patients who received neoadjuvant chemotherapy; (2) patients with additional malignancies; (3) bilateral lesions; (4) non-mass lesions with no delineation of boundaries. To validate the model, an external validation cohort was independently collected from the Third Affiliated Hospital of Harbin Medical University (Institution 2) between 2020 and 2021, following the same inclusion and exclusion criteria. Finally, we enrolled 420 patients and randomly assigned them to the training (n = 294) and test sets (n=126) using a ratio of 7:3. Five-fold cross-validation was performed on the training set, with the test set and external validation cohort (n=90, Institution 2) reserved for final evaluation (**Figure 2**).

According to St. Gallen risk criteria, patients were classified into high-risk and low-medium risk categories based on axillary lymph node status and molecular characteristics. Patients classified as low-to-medium risk were considered to have similar treatment regimens and clinical outcomes (5). Patients allocated to the high-risk category had ≥4 positive axillary nodes (≥4 N+) or 1–3 positive axillary nodes (1–3 N+) and were either estrogen receptor (ER−)/progesterone receptor (PR−) or HER2+. Patients allocated to the low-to-medium risk category were node-negative or had 1–3 positive nodes (1–3 N+) and were ER+ and/or PR+ and HER2− (24).

### 2.2 Clinicopathological characteristics collection

Clinical variables included age. The pathological and immunohistochemical variables included the following: tumor size, tumor histologic grade, lymph node metastasis, vascular invasion, ER, PR, and HER2. An ER- or PR-positive expression label was required for at least 1% nuclear staining in the tumor for immunohistochemistry (25). A HER2 positive expression diagnosis required a HER2 score of 3+ or 2+ and a positive fluorescence *in situ* hybridization (FISH) result (26); a value of 0 or 1+ was regarded as HER2 negative.

Breast cancers were classified into distinct molecular subtypes based on the expression of the ER, PR, and HER2: luminal A (HR-positive/HER2-negative), luminal B (HR-positive/HER2 positive), which expresses at least one HR, HER2, and has high levels of the Ki-67 protein, HER2-enriched (HR-negative/HER2-positive), and basal-like breast cancer (HR-negative/HER2-negative) (27).

### 2.3 Ultrasound Image acquisition and image segmentation

Ultrasound machines have been used for all examinations, including HIVISION AVIUS (Hitachi, Japan) and Aixplorer (SuperSonic Imagine, France), with corresponding high-frequency (5–12 MHz) probes. Obtaining two-dimensional images of the largest transverse and long-axis cross sections is customarily necessary. Two radiologists with more than 5 years of combined experience analyzed all ultrasound images. They were not involved in image acquisition and were unaware of the final diagnosis of each patient. If there were any discrepancies, the two experts discussed them and reached a consensus. The ACR BI-RADS Atlas (2013) was used to analyze the two-dimensional (2D) ultrasound images (28). The BI-RADS ultrasound features were included as follows: shape, orientation, margin, posterior acoustic pattern, calcification, echo pattern. Additionally, we also gained the Adler grade (0, 1, 2, and 3) (29), which is a measure of the blood flow determined using vascular distribution.

Two radiologists with a free, open-source imaging platform (3D Slicer 4.8.1, https://www.slicer.org) performed manual segmentation of ultrasound images. Only the largest of the multiple lesions was sketched, and the region of interest (ROI) was manually segmented along the entire tumor region, layer by layer. Since this study was to acquire images for two different machines, preprocessing was essential at the initial stage of feature extraction to address potential heterogeneity introduced by differences in imaging parameters, hardware characteristics, and image acquisition protocols. To minimize variability and ensure consistency, we implemented a standardized preprocessing pipeline that included resampling, intensity normalization, and noise reduction.

Using an eight-level quantization representation (30), the acquisition area was resampled using a trilinear interpolation algorithm to achieve a constant inclination and a specific isotropic resolution (voxel size = 1×1×1 mm³). Intensity normalization was performed by linearly scaling pixel values to a fixed range (0–255) to harmonize intensity distributions across images from both devices. Additionally, a Gaussian smoothing filter (kernel size = 3×3×3, σ = 1) was applied to reduce noise while preserving image texture features during preprocessing.

### 2.4 Radiomics features extraction, selection, and model development

#### 2.4.1 Radiomics features extraction

The extracted radiomics features include the following: (a) shape-based features; (b) first-order statistics features; (c) gray level cooccurrence matrix-based features (GLCM); (d) gray level run-length matrix-based features (GLRLM); (e) gray level size zone matrix (GLSZM); (f) neighboring gray tone difference matrix (NGTDM); (g) gray level dependence matrix (GLDM); and (h) transform-filtered features (including square, square root, logarithm, exponential, gradient, Laplacian of Gaussian, wavelet). The feature extraction was performed in Pyradiomics (v.3.0.1; https://www.python.org).

#### 2.4.2 ICC assessment

The intra-class correlation coefficient (ICC) was used to calculate the concordances between inter-observers in order to assess the consistency of radiomics features. To assess interobserver agreement, two radiologists carried out the segmentation process independently at the same time. An ICC score of more than 0.75 suggested that the ROIs were in good agreement.

#### 2.4.3 Radiomics features selection

Prior to feature selection, each radiomics feature was standardized to achieve a normal distribution and enhance discrimination between different data sets. Initially, features with ICCs below 0.75 were excluded to prevent redundancy. Subsequently, redundant and irrelevant features were eliminated using the mRMR algorithm. Finally, LASSO regression analysis was used to further select the most significant features. The tuning parameter (lambda) in the LASSO model was determined using 10-fold cross-validation. Specifically, lambda was chosen based on the “1-SE rule” (one standard error rule), which selects the most parsimonious model within one standard error of the lambda value that minimizes the cross-validation error. Features with non-zero coefficients were selected with the optimal lambda for further analysis.

#### 2.4.4 Radiomics model development and Rad-Score calculation

Efficient features were utilized to develop machine learning models employing eight classifiers, namely logistic regression (LR), gradient boosting tree (GBT), artificial neural network (ANN), support vector machine (SVM), random forest (RF), Naive Bayes (NB), k-nearest neighbor (KNN) and adaptive boosting (AdaBoost) to differentiate high recurrence risk from low-medium recurrence risk using the training set. Hyperparameters for all machine learning models were tuned via grid search with 5-fold cross-validation to optimize performance: LR (C=1.0), GBT (distribution = ‘bernoulli’, n.trees = 2000, shrinkage = 0.01), ANN (size=4,decay=5e-4,maxit=200), SVM (kernel=‘radial’,cost=1, gamma=0.1), RF (n.trees = 500, mtry = sqrt(ncol(train1) - 1), importance = TRUE), NB (var_smoothing=default value), KNN (k=tuned during grid search), AdaBoost (n.trees = 2000). The one with the best diagnostic performance was selected to develop the final radiomics model and then calculate the Radiomics score (Rad-score).

### 2.5 Nomogram construction

#### 2.5.1 Feature selection using logistic regression

Age as one of the clinical features was used as a covariate in our column chart. In the training set, BI-RADS ultrasound features associated with the risk of breast cancer recurrence were identified using univariate and multivariate logistic regression analysis. The features involved a backward-stepwise multivariate logistic regression to quantify the effect of Rad-score, age, and ultrasound risk informative features in predicting breast cancer recurrence risk. Collinearity was taken into consideration, and risk variables with *p* > 0.05 were disregarded.

#### 2.5.2 Model development and nomogram construction

To systematically evaluate the predictive performance of different feature sets, we developed three different models based on statistically significant clinical, BI-RADS ultrasound features and the Rad-score: (1) Clin-US model, (2) Clin-Rad model, and (3) Clin-US-Rad model. Each model was constructed and validated to identify the optimal feature combination for predicting breast cancer recurrence risk.

### 2.6 Nomogram evaluation

The ROC curve was applied to evaluate the discrimination performance of different models. To compare the AUC values of these models and assess whether their differences were statistically significant, the DeLong test was performed. The Hosmer-Lemeshow test was used to assess the nomogram’s goodness of fit. The calibration curve was used to evaluate the nomogram’s predictive accuracy regarding the agreement between the predicted probability and the actual probability of breast cancer recurrence. The net benefits at various threshold values were calculated using a decision curve analysis (DCA) to assess the therapeutic usefulness of the nomogram. Additionally, the accuracy, sensitivity, specificity, positive predictive value (PPV), and negative predictive value (NPV) were also calculated.

### 2.7 Statistical analysis

Statistical analyses were performed using SPSS (Version 25.0, IBM) and R software (Version 4.4.3, https://www.r-project.org). The t-test or Mann–Whitney U test was used to compare quantitative variables. Categorical variables between the training and test groups were compared using the Chi-square or Fisher’s test. Furthermore, multiple logistic regression analysis with a forward stepwise selection was used to identify a significant signature for predicting the high risk of recurrence. Odds ratios (ORs) with relative 95% confidence intervals (CIs) were calculated to determine the relevance of all potential predictors for the high risk of recurrence.

We used the ‘glmnet’ package for the LASSO logistic regression study. The ‘pROC’ software package was used to analyze ROC curves and perform the DeLong test to compare the AUC values of different models for statistical significance. Moreover, the nomogram and calibration curves were generated using the ‘rms’ package. The Hosmer–Lemeshow test and DCA were performed using the ‘generalhoslem’ and ‘rmda’ packages, respectively. *p* < 0.05 was considered statistically significant.

## 3 Results

### 3.1 Baseline characteristics

The baseline characteristics of the patients in the training, test, and external validation sets are shown in **Table 1**. Overall, 420 patients were included in this study. The patients were randomly divided into the training set (n = 294) and the test set (n = 126) at a ratio of 7:3. Additionally, an external validation set consisting of 90 patients from a separate cohort was included to validate the generalizability of the model. No statistically significant differences were observed in clinicopathological or two-dimensional BI-RADS ultrasound features among the training, test, and external validation sets.

**Table 1:** Baseline characteristics of patients in the training, test, and external validation cohorts.

Characteristics	Total (n=420)	Training set (n=294)	Test set (n=126)	External validation set (n=90)	*p*
Age (y)					0.245
<50	166 (39.5)	122 (41.5)	44 (34.9)	30 (33.3)	
≥50	254 (60.5)	172 (58.5)	82 (65.1)	60 (66.7)	
Tumor size (cm)					0.591
<2	198 (47.1)	134 (45.6)	64 (50.8)	44 (48.9)	
≥2	222 (52.9)	160 (54.4)	62 (49.2)	46 (51.1)	
Molecular subtypes					0.969
Luminal A	190 (45.2)	129 (43.9)	61 (48.4)	41 (45.6)	
Luminal B	111 (26.4)	79 (26.9)	32 (25.4)	24 (26.7)	
HER2-enriched	67 (16.0)	50 (17.0)	17 (13.5)	12 (13.3)	
Basal-like	52 (12.4)	36 (12.2)	16 (12.7)	13 (14.4)	
Tumor histologic grade					0.937
I	27 (6.4)	17 (5.8)	10 (7.9)	6 (6.67)	
II	211 (50.2)	150 (51)	61 (48.4)	44 (48.9)	
III	182 (43.4)	127 (43.2)	55 (43.7)	40 (44.4)	
Estrogen receptor					0.444
Positive	127 (30.2)	94 (32)	33 (26.2)	64 (71.1)	
Negative	293 (69.8)	200 (68)	93 (73.8)	26 (28.9)	
Progesterone receptor					0.690
Positive	152 (36.2)	104 (35.4)	48 (38.1)	54 (60.0)	
Negative	268 (63.8)	190 (64.6)	78 (61.9)	36 (40.0)	
HER2					0.630
Positive	269 (64)	184 (62.6)	85 (67.5)	33 (36.7)	
Negative	151 (36)	110 (37.4)	41 (32.5)	57 (63.3)	
Lymph node metastasis					0.176
Yes	232 (55.2)	162 (55.1)	70 (55.6)	50 (55.6)	
No	188 (44.8)	132 (44.9)	56 (44.4)	40 (44.4)	
Vascular invasion					0.274
Yes	372 (88.6)	263 (89.5)	109 (86.5)	15 (16.7)	
No	48 (11.4)	31 (10.5)	17 (13.5)	75 (83.3)	
Tumor shape					0.993
Round, oval	14 (3.3)	10 (3.4)	4 (3.2)	3 (3.33)	
Irregular	406 (96.7)	284 (96.6)	122 (96.8)	87 (96.7)	
Growth orientation					0.785
Parallel	305 (72.6)	211 (71.8)	94 (74.6)	23 (25.6)	
Vertical	115 (27.4)	83 (28.2)	32 (25.4)	67 (74.4)	
Margin					0.617
Circumscribed	23 (5.5)	14 (4.8)	9 (7.1)	5 (5.56)	
Indistinct	397 (94.5)	280 (95.2)	117 (92.9)	85 (94.4)	
Posterior acoustic pattern					0.705
No-shadowing	276 (65.7)	190 (64.6)	86 (68.3)	57 (63.3)	
Shadowing	144 (34.3)	104 (35.4)	40 (31.7)	33 (36.7)	
Calcification					0.826
Absent	228 (54.3)	159 (54.1)	69 (54.8)	52 (57.8)	
Present	192 (45.7)	135 (45.9)	57 (45.2)	38 (42.2)	
Echo pattern					0.320
Hypoechoic	350 (83.3)	248 (84.4)	102 (81)	70 (77.8)	
Heterogeneous	70 (16.7)	46 (15.6)	24 (19)	20 (22.2)	
Adler grade					0.583
0-1	191 (45.5)	132 (44.9)	59 (46.8)	46 (51.1)	
2-3	229 (54.5)	162 (55.1)	67 (53.2)	44 (48.9)	
BI-RADS grade					0.802
3-4b	178 (42.4)	127 (43.2)	51 (40.5)	36 (40.0)	
4c-5	242 (57.6)	167 (56.8)	75 (59.5)	54 (60.0)	
Risk of recurrence					0.921
Low-medium risk	282 (67.1)	197 (67)	85 (67.5)	50 (55.6)	
High risk	138 (32.9)	97 (33)	41 (32.5)	40 (44.4)	

y, years old; HER2, human epidermal growth factor receptor 2; BI-RADS, Breast Imaging-Reporting and Data System.

### 3.2 Clinical and BI-RADS ultrasound features selection

In the training set, univariate and multivariate logistic regression analyses were performed. The results showed that margin, echo pattern, and BI-RADS grade were significant BI-RADS ultrasound features for differential diagnosis (*p*<0.05) (**Table 2**).

**Table 2:** Univariate and Multivariate Logistic regression analysis in the training set.

Characteristics	Univariate analysis		Multivariate analysis	
	OR (95%CI)	*p*	OR (95%CI)	*p*
Age				
(≥50 vs.<50)	1.313(0.797-2.162)	0.285		
Tumor shape				
(Round, oval vs. Irregular)	2.011(0.419-9.654)	0.383		
Growth orientation				
(Parallel vs. Vertical)	0.822(0.482-1.401)	0.471		
Margin				
(Smooth vs. Lobulate)	3.081(0.676-14.049)	0.146		
Boundary				
(Circumscribed vs. Indistinct)	7.131(2.962-17.169)	<0.001*	4.685(1.612-13.612)	0.005*
Posterior echo				
(No attenuation vs. Attenuation)	1.775(1.075-2.932)	0.025		
Calcification				
(Absent vs. Present)	1.164(0.715-1.895)	0.541		
Internal echo				
(Hypoechoic vs. Mixed-echoic)	0.376(0.168-0.842)	0.017*	0.222(0.070-0.703)	0.010*
CDFI level				
(0-1 vs. 2-3)	0.670(0.411-1.093)	0.109		
BI-RADS grade				
(3-4b vs. 4c-5)	3.598(2.086-6.206)	<0.001*	3.689(1.787-8.739)	0.001*
Rad-score	5.130(3.341-7.877)	<0.001*	0.191(0.113-0.324)	<0.001*

Data in brackets are the 95% confidence intervals.

*p < 0.05 indicates that the predictive variable is independently associated with High risk of breast cancer recurrence.

### 3.3 Radiomics features extraction and selection

**Figure 3:**
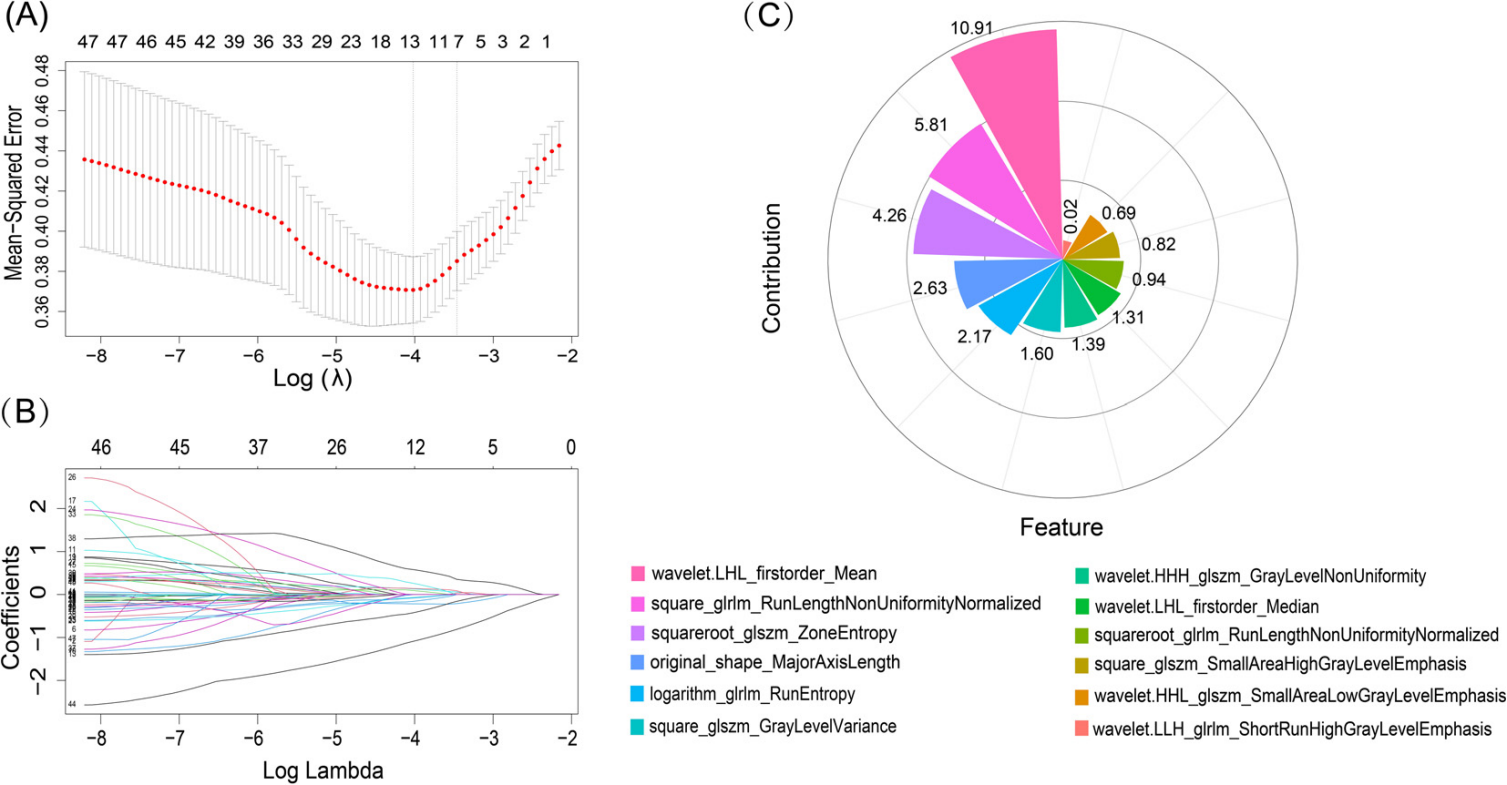
LASSO algorithm for radiomics features selection and features contribution coefficients. **(A)** The mean-squared error log was plotted (lambda). Dotted vertical lines were drawn at the optimal values using the minimum criteria and one standard error of the minimum criteria. The tuning parameter for the LASSO model was determined using 10-fold cross-test and minimal standards. **(B)** LASSO coefficient profiles of the 50 radiomics features. 12 features with nonzero coefficients were chosen to form the radiomics signature after the coefficients (y-axis) were plotted against log (lambda). **(C)** Features and their respective contribution coefficients in process of constructing radiomics model based on training set.

A total of 1314 features were extracted from each ROI in the US images. The entire collection of features comprised 14 shape-based, 18 first-order, 24 GLCM, 16 GLRLM, 16 GLSZM, 14 GLDM, 5 NGTDM features, and 1027 features derived from wavelet filter images. Subsequently, ICC analysis was performed, 420 features with an ICC value over 0.75 were chosen for radiomics modelling. Then, using the mRMR algorithm 100 features were retained. Finally, the 12 non-zero coefficient features were produced in feature selection using the LASSO method. The process of features selection and the selected 12 features with their contribution coefficients are shown in **Figure 3**.

### 3.4 Develop a radiomics model and construct Rad-score

**Figure 4:**
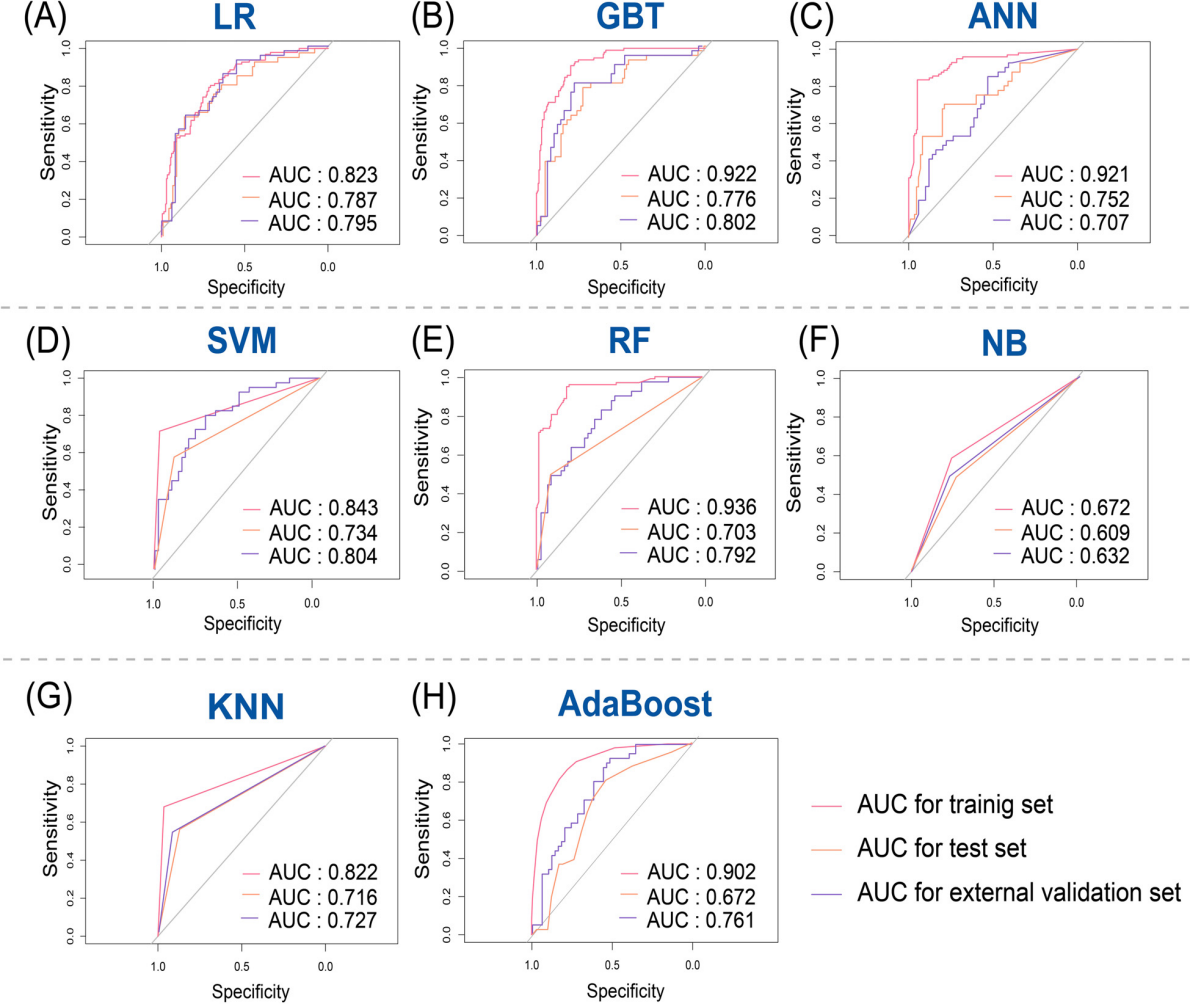
Receiver operating characteristic (ROC) curves for predicting breast cancer recurrence risk in training, test, and external validation sets. **(A)** Logistic regression (LR); **(B)** Gradient boosting tree (GBT); **(C)** Artificial neural network (ANN); **(D)** Support vector machine (SVM); **(E)** Random Forest (RF); **(F)** Naive Bayes (NB); **(G)** K-nearest neighbor (KNN); **(H)** Adaptive Boosting (AdaBoost).

According to the selected features, eight different machine learning models including LR, GBT, ANN, SVM, RF, NB, KNN and AdaBoost were used to construct the radiomics model, and the ROC were used to evaluate the different models’ predictive ability. Both the test set and external validation set demonstrated that the LR model performed best among the eight models, which were used to build the final radiomics model. Feature importance rankings for RF, GBT, and AdaBoost, the top-performing tree-based models, are provided in **Supplementary Figure 3**. The radiomics model yielded AUC values of 0.823 (95% CI: 0.774–0.871), 0.787 (95% CI: 0.704–0.873), and 0.795 (95% CI: 0.674–0.880) in the training, test, and external validation sets, respectively (**Figure 4**).

**Figure 5:**
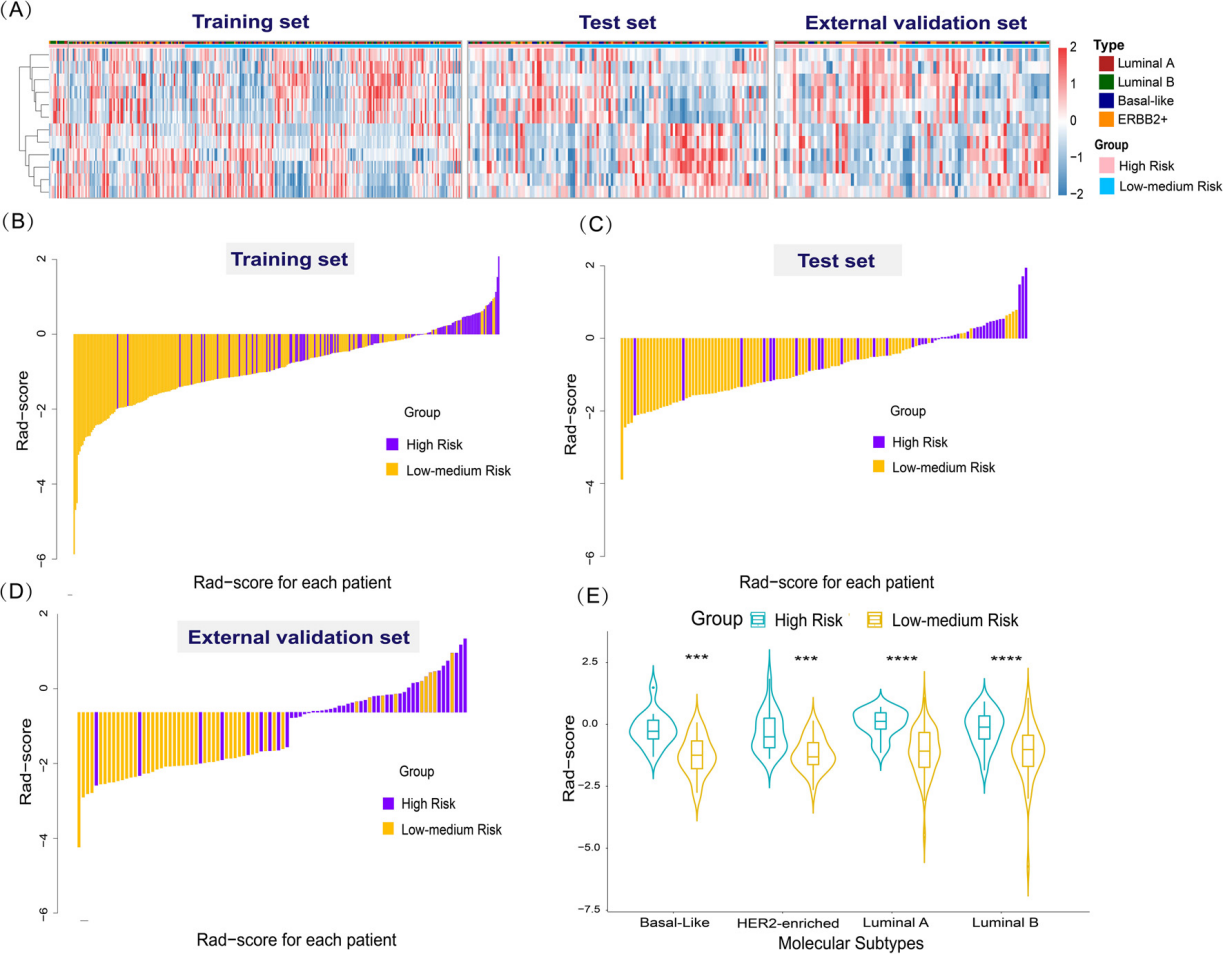
Radiomics features and performance of the radiomics score. **(A)** Heat map of 12 selected radiomics features. (Separately grouped for the training, test, and external validation sets and the high-risk group vs. low to medium risk group). **(B–D)** The radiomics score for each patient in the training, test, and external validation sets. **(E)** Violin plot of the role of Rad-score in predicting the risk of breast cancer recurrence in different molecular subtypes. Violin plot of the role of Rad-score in predicting the risk of breast cancer recurrence in different molecular subtypes (***p < 0.001,****p < 0.0001).

The radiomics model’s results were used to calculate the Rad-score, which was significantly higher in the high-risk of recurrence group than in the low-to-medium-risk of recurrence group in the training set (-0.17 ± 0.70 vs. -1.20 ± 0.95, *p*<0.001), the test set (-0.13 ± 0.83 vs. -1.09 ± 0.79, *p*<0.001), and the external validation set (-0.18 ± 0.74 vs. -1.21 ± 0.89, *p*<0.001). The distributions of recurrence risk Rad-score and outcomes for each patient in the training, test, and external validation sets are shown in **Figures 5A–D**. To further validate the generality and applicability of our developed Rad-score, we performed studies in different molecular subtypes of breast cancer. As shown in **Figure 5E**, among the different molecular subtypes of breast cancer, the radiomics score was also significantly higher in the high-risk recurrence group than in the low-to-medium-risk recurrence group. This suggests that our constructed Rad-score can be used to predict the risk of recurrence in four different breast cancer subtypes.

### 3.5 Nomograms construction and effectiveness evaluation

**Figure 6:**
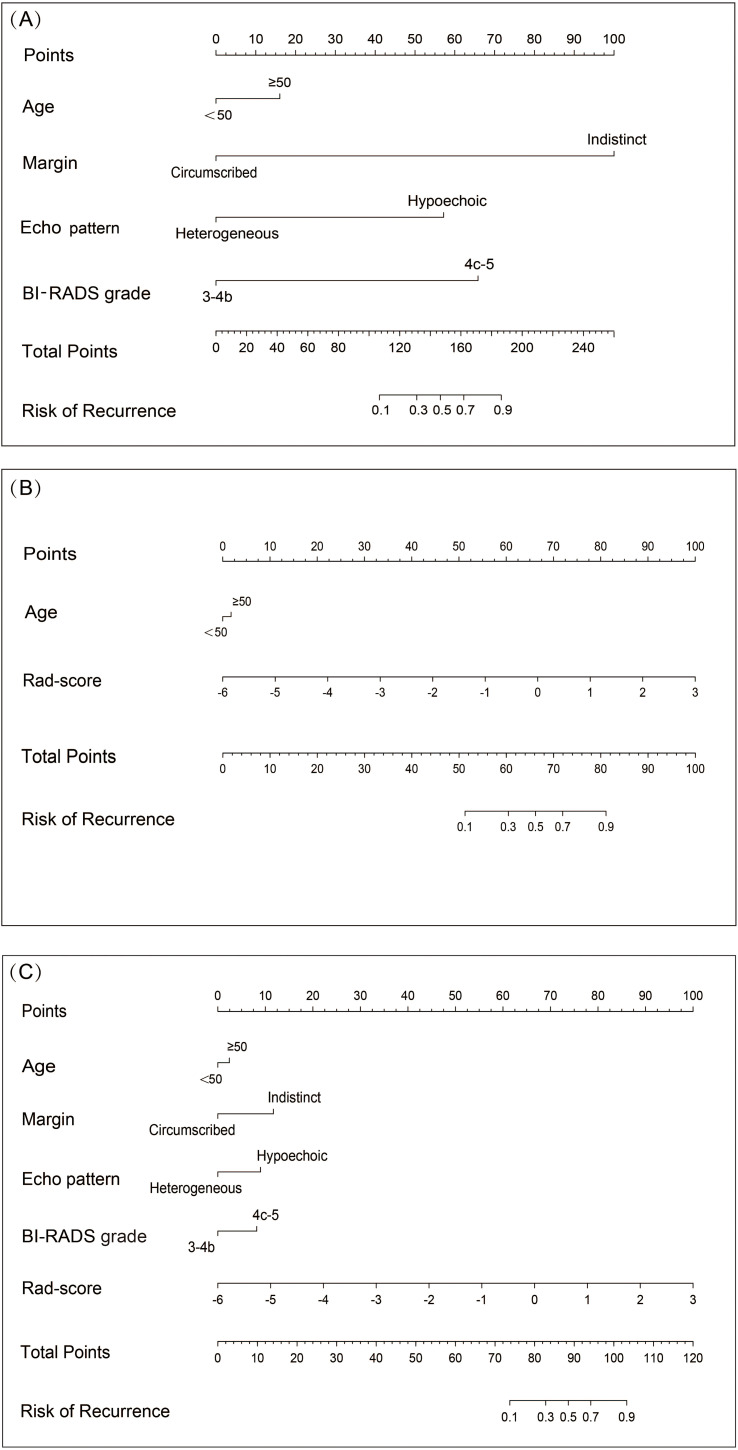
Nomograms of different models for predicting breast cancer recurrence risk. **(A)** Nomogram plots of Clin-US model for predicting breast cancer recurrence risk. **(B)** Nomogram plots of Clin-Rad model for predicting breast cancer recurrence risk. **(C)** Nomogram plots of Clin-US-Rad model for predicting breast cancer recurrence risk.

We constructed three distinct diagnostic nomogram models using the informative features identified in this study: the Clin-US, the Clin-Rad, and the combined (Clin-US-Rad) models (**Figure 6**).

**Figure 7:**
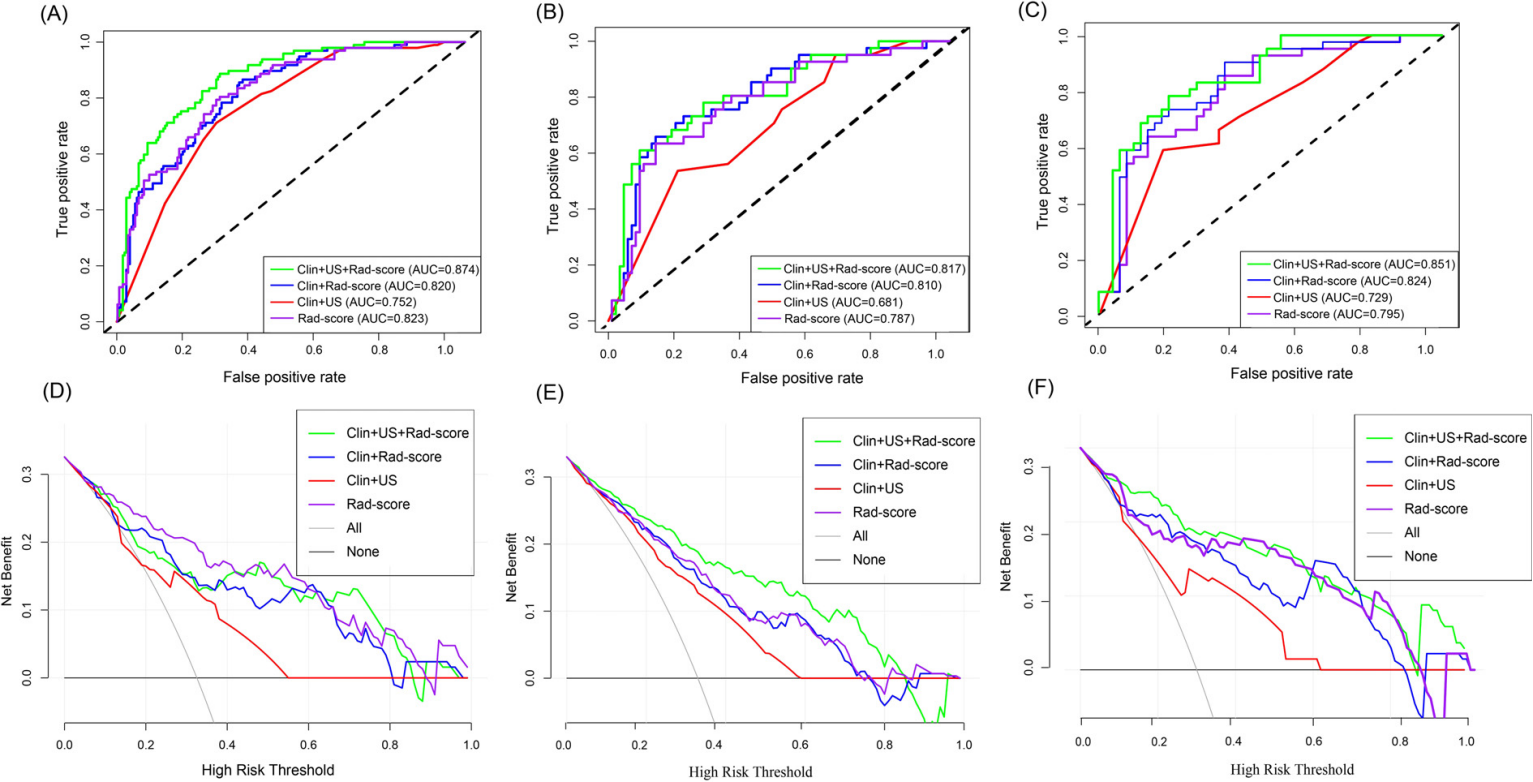
Diagnostic performance of different models to predict the risk of breast cancer recurrence. AUC plots of different models predicting risk of breast cancer recurrence in the training set **(A)**, test set **(B)**, and external validation set **(C)**. Decision curves of different models for predicting breast cancer recurrence risk in the training set **(D)**, test set **(E)**, and external validation set **(F)**.

In the training set, the Clin-US, Clin-Rad, and combined models yielded AUC values of 0.752 (95% CI: 0.695 – 0.809), 0.820 (95% CI: 0.771 – 0.868), and 0.874 (95% CI: 0.833 – 0.943), respectively. In the test set, the Clin-US, Clin-Rad, and combined models yielded AUC values of 0.681 (95% CI: 0.619-0.812), 0.810(95% CI: 0.747-0.904), and 0.817 (95% CI: 0.762-0.912), respectively. In the external validation set, the Clin-US, Clin-Rad, and combined models yielded AUC values of 0.729 (95% CI: 0.623-0.815), 0.824 (95% CI: 0.704-0.849), and 0.851(95% CI: 0.754-0.916), respectively. The DeLong test revealed that the combined model significantly outperformed the other models in all sets (*p*<0.05) (**Supplementary Figure 1**). As shown in **Figure 7A**, the combined model presented optimal performance and the best AUC value in the training set. Similarly, the combined model also demonstrated the highest AUC in the test set (**Figure 7B**) and the external validation set (**Figure 7C**). Meanwhile, the accuracy of the combined model was 80.6%, 78.6%, and 76.7% in the training, test, and external validation sets, respectively (**Table 3**).

**Table 3:** Performance of different models for predicting breast cancer recurrence risk in training, test, and external validation sets.

Models	Cohorts	AUC (95%CI)	ACC (95%CI)	Sensitivity (95%CI)	Specificity (95% CI)	PPV (95% CI)	NPV (95% CI)
Radiomics model	training	0.823 (0.774-0.871)	0.752 (0.699-0.798)	0.650 (0.541-0.745)	0.790 (0.730-0.839)	0.536 (0.437-0.632)	0.858 (0.802-0.899)
	test	0.787 (0.704-0.873)	0.778 (0.698-0.842)	0.686 (0.520-0.815)	0.813 (0.721-0.879)	0.585 (0.434-0.722)	0.871 (0.783-0.926)
	external validation	0.795 (0.674-0.880)	0.733 (0.622-0.811)	0.585 (0.413-0.733)	0.857 (0.714-0.935)	0.774 (0.580-0.706)	0.711 (0.609-0.818)
Clin-US model	training	0.752 (0.695-0.809)	0.707 (0.653-0.757)	0.649 (0.550-0.737)	0.736 (0.670-0.793)	0.548 (0.457-0.636)	0.810 (0.746-0.861)
	test	0.681 (0.619-0.812)	0.611 (0.524-0.692)	0.561 (0.410-0.701)	0.635 (0.529-0.730)	0.426 (0.303-0.558)	0.750 (0.639-0.836)
	external validation	0.729 (0.623-0.815)	0.711 (0.623-0.784)	0.605 (0.619.-0.717)	0.816 (0.679.-0.806)	0.727 (0.666.-0.816)	0.701 (0.672.-0.803)
Clin-Rad model	training	0.820 (0.771-0.868)	0.762 (0.710-0.807)	0.546 (0.447-0.642)	0.868 (0.814-0.908)	0.795 (0.736-0.844)	0.671 (0.561-0.764)
	test	0.810 (0.747-0.904)	0.810 (0.732-0.869)	0.585 (0.434-0.722)	0.917 (0.840-0.960)	0.821 (0.732-0.885)	0.774 (0.602-0.886)
	external validation	0.824 (0.704-0.849)	0.767 (0.659-0.844)	0.625 (0.523-0.620)	0.868 (0.805-0.892)	0.857 (0.666-0.878)	0.725 (0.629-0.828)
Clin-US-Rad model	training	0.874 (0.833–0.943)	0.806 (0.757-0.847)	0.639 (0.534-0.728)	0.888 (0.837-0.925)	0.833 (0.777-0.878)	0.738 (0.635-0.820)
	test	0.817 (0.762-0.912)	0.786 (0.706-0.848)	0.610 (0.457-0.743)	0.871 (0.783-0.926)	0.822 (0.731-0.886)	0.694 (0.531-0.820)
	external validation	0.851 (0.754-0.916)	0.767 (0.644-0.833)	0.675 (0.506-0.709)	0.858 (0.810-0.926)	0.857 (0.740-0.905)	0.726 (0.601-0.823)

AUC, Area under the ROC; ACC, Accuracy; PPV, Positive predictive value; NPV, Negative predictive value.

According to the DCA curve in **Figures 7D–F**, the combined model has a larger area under the curve and a net benefit over a “ all treated “ or “no treated “ strategy in the threshold probability range of 0.1-1.0. The Clin-US-Rad model had the highest net benefit at a threshold of =40%, demonstrating its clinical value in optimizing treatment decisions. For example, a 55-year-old patient (with a 45% predicted risk of recurrence) was advised to receive chemotherapy, while a 45-year-old patient (with a 30% predicted recurrence risk) avoided unnecessary chemotherapy. This suggests that the model improves clinical benefit while reducing overtreatment.

**Figure 8:**
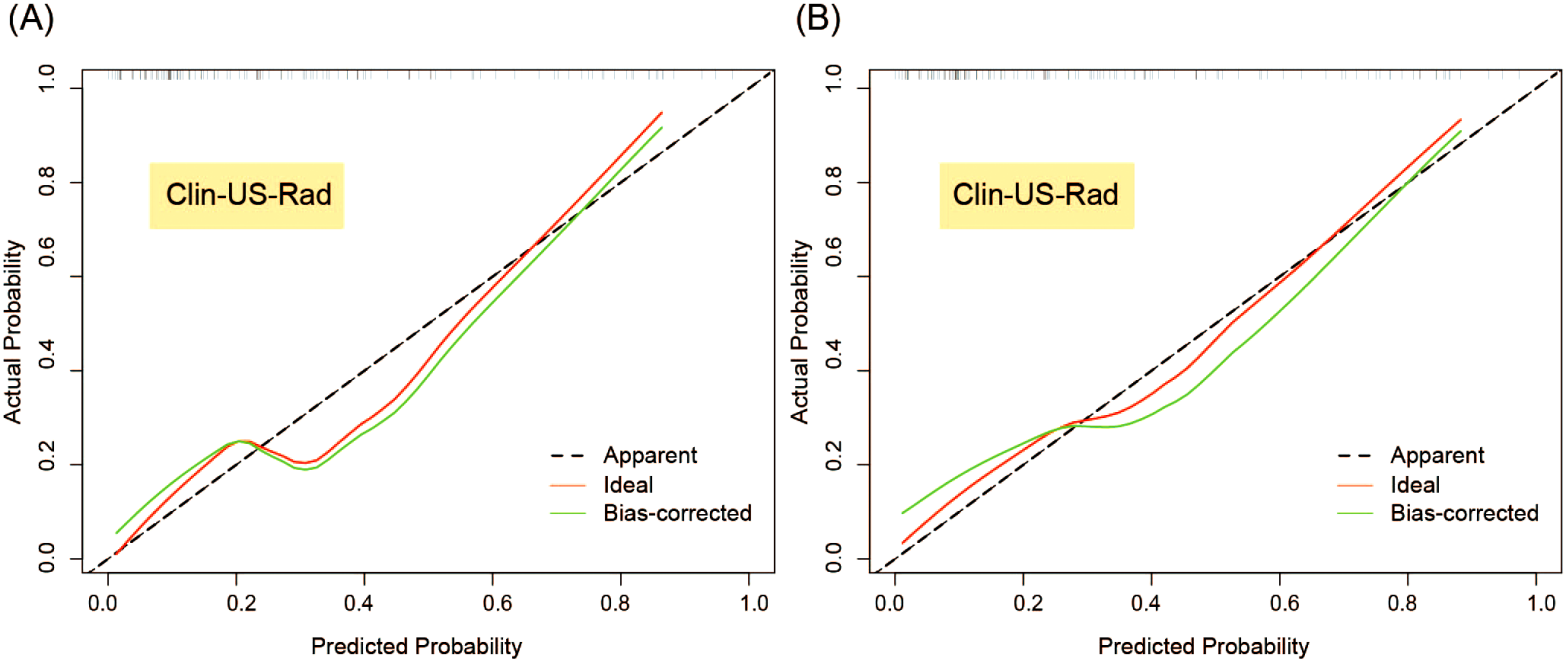
Calibration curves of Clin-US-Rad model for predicting breast cancer recurrence risk in the test **(A)** and external validation sets **(B)**.

The calibration curve of the combined model in the test set and the Hosmer-Lemeshow test demonstrated that the bias curve closely aligned with the ideal curve, indicating strong agreement between model predictions and observations. Similar results were observed in the external validation set, further supporting the robustness of the combined model (**Figure 8**). For completeness, the calibration curves of the other models in the test set and external validation set are provided in **Supplementary Figure 2**, which also exhibit favorable calibration performance.

## 4 Discussion

Breast cancer is a genetically heterogeneous disease with significant differences in recurrence risk even after standardized treatment (31). Therefore, preoperative noninvasive predicting the recurrence risk of breast cancer is crucial. Here, we developed the radiomics models based on breast ultrasound images for predicting the recurrence risk of breast cancer. Our study demonstrated that radiomics nomograms exhibited good performance in predicting the risk of breast cancer recurrence, and they could more conveniently individualize the prediction of breast cancer recurrence risk for patients preoperatively.

In this study, radiomics features within breast cancer tumors were extracted based on two-dimensional ultrasound images, 12 of which were associated with the risk of recurrence. The significant 12 ultrasound radiomics features were considered independent prognostic indicators in the training, test, and external validation sets. Among them, it is mainly composed of wavelet transform features and contains only one shape-based original feature. Wavelet transform features have proven highly effective in capturing tumor heterogeneity, which is closely related to cancer progression and recurrence (32, 33). Specifically, features such as wavelet.LHL reflect fine-grained variations in tissue texture and structure. These variations are often indicative of underlying biological processes, including cellular proliferation, angiogenesis, and intra-tumoral necrosis, which are key hallmarks of tumor heterogeneity (34, 35). For instance, Yu et al. (36) predicted the local recurrence of triple-negative breast cancer. They discovered that the majority of the 32 extracted radiomics features came from the wavelet transform features, which more accurately reflected tumor heterogeneity and was in line with our findings. These features’ ability to capture intricate structural patterns makes them superior predictors of recurrence risk. Additionally, entropy, another important radiomics feature, quantifies internal pixel distribution heterogeneity and has been established as a strong predictor of recurrence in primary breast cancer patients (37). Taken together, our findings suggest that wavelet transform features play an important role in reflecting tumor heterogeneity and predicting the risk of recurrence in breast cancer patients.

As an important indicator of breast cancer recurrence risk, age has been reported in many studies. The risk of recurrence increases significantly with increasing patient age (38, 39). Therefore, in this study, age was included as a covariate in the model to investigate its effect. Margin indistinct, echo pattern hypoechoic and higher BI-RADS grade were independent risk features for breast cancer recurrence. BI-RADS ultrasound features with indistinct margins suggest that breast cancer may rapidly grow and infiltrate into surrounding tissues (40). The more pronounced this feature means that cancer cells are more aggressive. Echo pattern hypoechoic may be due to its rapid proliferation, and it has been shown (41) that breast cancer cells have a high mitotic rate, resulting in the production of a large number of cancer cells, which destroys the internal normal tissue structure and changes echogenicity. This active proliferative property is associated with the ability of the tumor to invade and metastasize, which in turn produces more cancer cells, resulting in hypo echogenicity inside the lesion. This finding is consistent with the results of this study. Finally, BI-RADS grading as an interpretive criterion for sonographers of breast nodules, its high grade represents a higher degree of malignancy, which in turn increases the risk of recurrence (42, 43). Taken together, our findings further confirm the importance of BI-RADS ultrasound features in the prediction of breast cancer recurrence and provide a deeper understanding of it.

We developed eight different machine learning models to evaluate the ability of ultrasound radiomics features, among which SVM, GBT, and RF can handle high-dimensional problems. However, a general solution for nonlinear problems is unavailable. NB only needs a few parameters, but there a large error rate exists because prior probabilities should be computed. KNN is easy to implement, but the uncertainty of the K value has a great impact on the data results. Finally, artificial neural network can process data on a large scale but the output results are challenging to interpret, which can affect the reliability and acceptability of the results. LR is highly interpretable, while regularization is used to prevent filtering against noise interference, but it is limited by the hypothesis of linearity between features and targets. In our study, we found that the LR classifier produced the largest AUC, indicating a linear association between ultrasound radiomics features and the risk of breast cancer recurrence. Simultaneously, we also analyzed the performance of the radiomics model developed using LR in predicting the risk of breast cancer recurrence in different molecular subtypes. Our model has also been found to work well.

In this study, we develop a radiomics model (Rad model) to predict the risk of breast cancer recurrence. Previous studies have shown that the application of a single marker has some limitations in predictive performance. Therefore, we adopted a strategy combining multiple markers to improve the prediction performance. Zhu et al. (44) used radiomics model based on two-dimensional ultrasound images and combined models based on ultrasound images and clinical information to predict Ki-67 expression in invasive ductal carcinoma of breast cancer, The basic model’s test set AUC was 0.710, while the combined model’s AUC increased to 0.770. Meanwhile, Du et al. (45) established a nomogram model for predicting lymph vascular invasion of invasive breast cancer based on ultrasound images and clinical parameters. The model’s AUC values in the internal and external validation test sets were, respectively, 0.890 and 0.954, which were significantly higher than those in the ultrasound-clinical model (AUC: 0.787, 0.804), and high prediction accuracy was achieved. Based on the above findings, we integrated radiomics, BI-RADS ultrasound, and clinical features into three nomogram-visualized models to identify the optimal feature combination for diagnosis. These nomograms are visually and quantitatively integrating different risk features to predict the risk of breast cancer recurrence. Take the Clin-US-Rad model as an example: a 68-year-old female with an indistinct, hypoechoic lesion, BI-RADS 4b, radiomics score 0. The above characteristics are classified into the risk stratification, and the corresponding scores are obtained: 5 points, 11 points, 10 points, 2 points, and 68 points. The total sum is 96 points, and the total corresponding recurrence risk probability is more than 70%. It is exciting that the results of our study showed that all three models performed better than the single radiomics model in prediction. In particularly, the combined model showed the best predictive effect, further confirming the importance and value of the combined model in breast cancer recurrence risk prediction.

There are still some limitations in this study that need to be discussed in depth: First, the retrospective design of patient selection may introduce selection bias, affecting the universality of the study results, so it needs to be further verified by prospective studies. Secondly, the small number of high-risk patients could limit the model’s generalizability, increase the risk of overfitting, and result in broader confidence intervals. In the future, it is necessary to expand the sample size to improve the reliability of the results. Then, in this study, only two-dimensional ultrasound image data were used to construct the model, and the potential information of multimodality ultrasound images was not considered. Finally, our study has no follow-up results, and a part of follow-up results should be added later. In general, expanding the sample size, introducing multi-modal data and supplementing follow-up results will be important directions for future research.

## 5 Conclusions

In conclusion, radiomics model based on breast ultrasound images are a promising imaging predictor for assessing the risk of breast cancer recurrence. The nomogram combining radiomics, BI-RADS ultrasound, and clinical features offers clinicians a cost-effective decision-making tool, achieving accuracy while reducing unnecessary procedures.

## Data Availability

The original contributions presented in the study are included in the article/**Supplementary Material**. Further inquiries can be directed to the corresponding authors.

## Supplementary material

Supplementary Figure 1 DeLong’s test was performed to compare the AUCs between different models in the test set **(A)** and external validation set **(B)**. (Model 1: Radiomics model; Model 2: Clin-US model; Model 3: Clin-Rad model; Model 4: Clin-US-Rad model)

Supplementary Figure 2 Calibration curves of different models for predicting breast cancer recurrence risk in the test **(A–C)** and external validation sets **(D–F)**.

Supplementary Figure 3 Importance ranking of key features in radiomics weighted bar charts of RF, GBT, and AdaBoost models.

## References

[1] Siegel RL Miller KD Fuchs HE Jemal A. Cancer statistics, 2022. CA Cancer J Clin. (2022) 72:7–33. doi: 10.3322/caac.21708 35020204

[2] Wang X Wang N Zhong L Wang S Zheng Y Yang B. Prognostic value of depression and anxiety on breast cancer recurrence and mortality: a systematic review and meta-analysis of 282,203 patients. Mol Psychiatry. (2020) 25:3186–97. doi: 10.1038/s41380-020-00865-6 PMC7714689 32820237

[3] Bushnell GG Deshmukh AP den Hollander P Luo M Soundararajan R Jia D. Breast cancer dormancy: need for clinically relevant models to address current gaps in knowledge. NPJ Breast Cancer. (2021) 7:66. doi: 10.1038/s41523-021-00269-x 34050189 PMC8163741

[4] Buus R Sestak I Kronenwett R Ferree S Schnabel CA Baehner FL. Molecular drivers of Oncotype DX, prosigna, EndoPredict, and the breast cancer index: A TransATAC study. J Clin Oncol. (2021) 39:126–35. doi: 10.1200/JCO.20.00853 PMC8078458 33108242

[5] Burstein HJ Curigliano G Thurlimann B Weber WP Poortmans P Regan MM. Customizing local and systemic therapies for women with early breast cancer: the St. Gallen International Consensus Guidelines for treatment of early breast cancer 2021. Ann Oncol. (2021) 32:1216–35. doi: 10.1016/j.annonc.2021.06.023 PMC9906308 34242744

[6] Nguyen VC Nguyen TQ Vu TNH Phung TH Nguyen TPH Nguyen ND. Application of St Gallen categories in predicting survival for patients with breast cancer in Vietnam. Cancer Control. (2019) 26:1073274819862794. doi: 10.1177/1073274819862794 31307207 PMC6636225

[7] Kunheri B Raj RV Vijaykumar DK Pavithran K. Impact of St. Gallen surrogate classification for intrinsic breast cancer sub-types on disease features, recurrence, and survival in South Indian patients. Indian J Cancer. (2020) 57:49–54. doi: 10.4103/ijc.IJC_437_18 31929235

[8] Rabaglio M Aebi S Castiglione-Gertsch M. Controversies of adjuvant endocrine treatment for breast cancer and recommendations of the 2007 St Gallen conference. Lancet Oncol. (2007) 8:940–9. doi: 10.1016/S1470-2045(07)70317-0 17913663

[9] Jokar N Velikyan I Ahmadzadehfar H Rekabpour SJ Jafari E Ting HH. Theranostic approach in breast cancer: A treasured tailor for future oncology. Clin Nucl Med. (2021) 46:e410–e20. doi: 10.1097/RLU.0000000000003678 34152118

[10] Guidelines for diagnosis and treatment of advanced breast cancer in China (2022 edition). 10.1016/j.jncc.2023.12.001 PMC11390704 39282589

[11] Portnow LA-O Kochkodan-Self JM Maduram A Barrios MA-OX Onken AM Hong X. Multimodality imaging review of HER2-positive breast cancer and response to neoadjuvant chemotherapy. Radiographics. (2023) 43:e220103. doi: 10.1148/rg.220103 36633970

[12] Luo Y Gao Y Niu Z Zhang J Liu Z Zhang Y. The added value of ultrasound imaging biomarkers to clinicopathological factors for the prediction of high-risk Oncotype DX recurrence scores in patients with breast cancer. Quant Imaging Med Surg. (2024) 14:3519–33. doi: 10.21037/qims-23-1620 PMC11074736 38720854

[13] Geisel J Raghu M Hooley R. The role of ultrasound in breast cancer screening: the case for and against ultrasound. Semin Ultrasound CT MR. (2018) 39:25–34. doi: 10.1053/j.sult.2017.09.006 29317037

[14] Hooley RJ Scoutt LM Philpotts LE. Breast ultrasonography: state of the art. Radiology. (2013) 268:642–59. doi: 10.1148/radiol.13121606 23970509

[15] Wang H Zhan W Chen W Li Y Chen X Shen K. Sonography with vertical orientation feature predicts worse disease outcome in triple negative breast cancer. Breast. (2020) 49:33–40. doi: 10.1016/j.breast.2019.10.006 31677531 PMC7375680

[16] Zhang L Zhang X Han P Zhao D Hu N Fan W. Nomograms predicting recurrence in patients with triple negative breast cancer based on ultrasound and clinicopathological features. Br J Radiology. (2022) 95:20220305. doi: 10.1259/bjr.20220305 PMC9815727 35819909

[17] Guo Q Zhang L Di Z Ning C Dong Z Li Z. Assessing risk category of breast cancer by ultrasound imaging characteristics. Ultrasound Med Biol. (2018) 44:815–24. doi: 10.1016/j.ultrasmedbio.2017.12.001 29331358

[18] Mayerhoefer ME Materka A Langs G Haggstrom I Szczypinski P Gibbs P. Introduction to radiomics. J Nucl Med. (2020) 61:488–95. doi: 10.2967/jnumed.118.222893 PMC9374044 32060219

[19] Conti A Duggento A Indovina I Guerrisi M Toschi N. Radiomics in breast cancer classification and prediction. Semin Cancer Biol. (2021) 72:238–50. doi: 10.1016/j.semcancer.2020.04.002 32371013

[20] Romeo V Cuocolo R Apolito R Stanzione A Ventimiglia A Vitale A. Clinical value of radiomics and machine learning in breast ultrasound: a multicenter study for differential diagnosis of benign and Malignant lesions. Eur Radiol. (2021) 31:9511–9. doi: 10.1007/s00330-021-08009-2 PMC8589755 34018057

[21] Zheng X Yao Z Huang Y Yu Y Wang Y Liu Y. Deep learning radiomics can predict axillary lymph node status in early-stage breast cancer. Nat Commun. (2020) 11:1236. doi: 10.1038/s41467-020-15027-z 32144248 PMC7060275

[22] Bene I Ciurea AI Ciortea CA Stefan PA Ciule LD Lupean RA. Radiomic signatures derived from hybrid contrast-enhanced ultrasound images (CEUS) for the assessment of histological characteristics of breast cancer: A pilot study. Cancers (Basel). (2022) 14. doi: 10.3390/cancers14163905 PMC9405598 36010897

[23] Wu J Zhang H Li L Hu M Chen L Xu B. A nomogram for predicting overall survival in patients with low-grade endometrial stromal sarcoma: A population-based analysis. Cancer Commun (Lond). (2020) 40:301–12. doi: 10.1002/cac2.12067 PMC7365459 32558385

[24] Goldhirsch A Wood WC Gelber RD Coates AS Thurlimann B Senn HJ. Progress and promise: highlights of the international expert consensus on the primary therapy of early breast cancer 2007. Ann Oncol. (2007) 18:1133–44. doi: 10.1093/annonc/mdm271 17675394

[25] Hammond ME Hayes DF Dowsett M Allred DC Hagerty KL Badve S. American Society of Clinical Oncology/College of American Pathologists guideline recommendations for immunohistochemical testing of estrogen and progesterone receptors in breast cancer. J Clin Oncol. (2010) 28:2784–95. doi: 10.1200/JCO.2009.25.6529 PMC2881855 20404251

[26] Wolff AC Hammond ME Hicks DG Dowsett M McShane LM Allison KH. Recommendations for human epidermal growth factor receptor 2 testing in breast cancer: American Society of Clinical Oncology/College of American Pathologists clinical practice guideline update. J Clin Oncol. (2013) 31:3997–4013. doi: 10.1200/JCO.2013.50.9984 24101045

[27] Perou CM Sorlie T Eisen MB van de Rijn M Jeffrey SS Rees CA. Molecular portraits of human breast tumours. Nature. (2000) 406:747–52. doi: 10.1038/35021093 10963602

[28] Sickles E D’Orsi C Bassett L. ACR BI-RADS^®^ Atlas: Breast Imaging Reporting and Data System Vol. 293. Reston, VA: American College of Radiology (2013).

[29] Adler DD Carson PL Rubin JM Quinn-Reid D. Doppler ultrasound color flow imaging in the study of breast cancer: preliminary findings. Ultrasound Med Biol. (1990) 16:553–9. doi: 10.1016/0301-5629(90)90020-d 2238263

[30] Wichtmann BD Harder FN Weiss K Schonberg SO Attenberger UI Alkadhi H. Influence of image processing on radiomic features from magnetic resonance imaging. Invest Radiol. (2022) 58:199-208. doi: 10.1097/RLI.0000000000000921 36070524

[31] Waks AG Winer EP. Breast cancer treatment: A review. JAMA. (2019) 321:288–300. doi: 10.1001/jama.2018.19323 30667505

[32] Wang X Xie T Luo J Zhou Z Yu X Guo X. Radiomics predicts the prognosis of patients with locally advanced breast cancer by reflecting the heterogeneity of tumor cells and the tumor microenvironment. Breast Cancer Res. (2022) 24:20. doi: 10.1186/s13058-022-01516-0 35292076 PMC8922933

[33] Kang W Qiu X Luo Y Luo J Liu Y Xi J. Application of radiomics-based multiomics combinations in the tumor microenvironment and cancer prognosis. J Transl Med. (2023) 21:598. doi: 10.1186/s12967-023-04437-4 37674169 PMC10481579

[34] Barzaman K Karami J Zarei Z Hosseinzadeh A Kazemi MH Moradi-Kalbolandi S. Breast cancer: Biology, biomarkers, and treatments. Int Immunopharmacol. (2020) 84:106535. doi: 10.1016/j.intimp.2020.106535 32361569

[35] Morales-Guadarrama G García-Becerra R Méndez-Pérez EA García-Quiroz J Avila E Díaz L. Vasculogenic mimicry in breast cancer: clinical relevance and drivers. Cells. (2021) 10. doi: 10.3390/cells10071758 PMC8304745 34359928

[36] Yu F Hang J Deng J Yang B Wang J Ye X. Radiomics features on ultrasound imaging for the prediction of disease-free survival in triple negative breast cancer: a multi-institutional study. Br J Radiol. (2021) 94:20210188. doi: 10.1259/bjr.20210188 34478336 PMC9328043

[37] Kim J-H Ko ES Lim Y Lee KS Han B-K Ko EY. Breast cancer heterogeneity: MR imaging texture analysis and survival outcomes. Radiology. (2017) 282:665–75. doi: 10.1148/radiol.2016160261 27700229

[38] Schaffar R Benhamou S Chappuis PO Rapiti E. Risk of first recurrence after treatment in a population-based cohort of young women with breast cancer. Breast Cancer Res Treat. (2024) 206:615–23. doi: 10.1007/s10549-024-07338-2 PMC11208255 38687430

[39] Daly MB Rosenthal E Cummings S Bernhisel R Kidd J Hughes E. The association between age at breast cancer diagnosis and prevalence of pathogenic variants. Breast Cancer Res Treat. (2023) 199:617–26. doi: 10.1007/s10549-023-06946-8 PMC10175307 37084156

[40] Huang Z Chen L Wang Y Fu L Lv R. Molecular markers, pathology, and ultrasound features of invasive breast cancer. Clin Imaging. (2021) 79:85–93. doi: 10.1016/j.clinimag.2021.03.039 33895560

[41] Au FW-F Ghai S Lu F-I Moshonov H Crystal P. Histological grade and immunohistochemical biomarkers of breast cancer: correlation to ultrasound features. J Ultrasound Med. (2017) 36:1883–94. doi: 10.1002/jum.14247 28556296

[42] Kubota K Iwasa H Aoyama N Hamada N Nogami M Tamura T. Power Doppler and gray-scale sonography standardized by BI-RADS for the differentiation of benign postoperative lesion and local recurrence after breast-conserving therapy. Oncol Rep. (2011) 26:1357–62. doi: 10.3892/or.2011.1445 21887473

[43] Gweon HM Son EJ Youk JH Kim J-A Chung J. Value of the US BI-RADS final assessment following mastectomy: BI-RADS 4 and 5 lesions. Acta Radiologica. (2012) 53:255–60. doi: 10.1258/ar.2011.110597 22302210

[44] Zhu Y Dou Y Qin L Wang H Wen Z. Prediction of Ki-67 of invasive ductal breast cancer based on ultrasound radiomics nomogram. J Ultrasound Med. (2023) 42:649–64. doi: 10.1002/jum.16061 35851691

[45] Du Y Cai M Zha H Chen B Gu J Zhang M. Ultrasound radiomics-based nomogram to predict lymphovascular invasion in invasive breast cancer: a multicenter, retrospective study. Eur Radiol. (2024) 34:136–48. doi: 10.1007/s00330-023-09995-1 37518678

