# Supplementary figures and images for: Machine learning-based ultrasound radiomics for predicting risk of recurrence in breast cancer
Source: Front Oncol. 2025 May 12;15:1542643. doi: 10.3389/fonc.2025.1542643 (PMC12104244; doi:10.3389/fonc.2025.1542643)

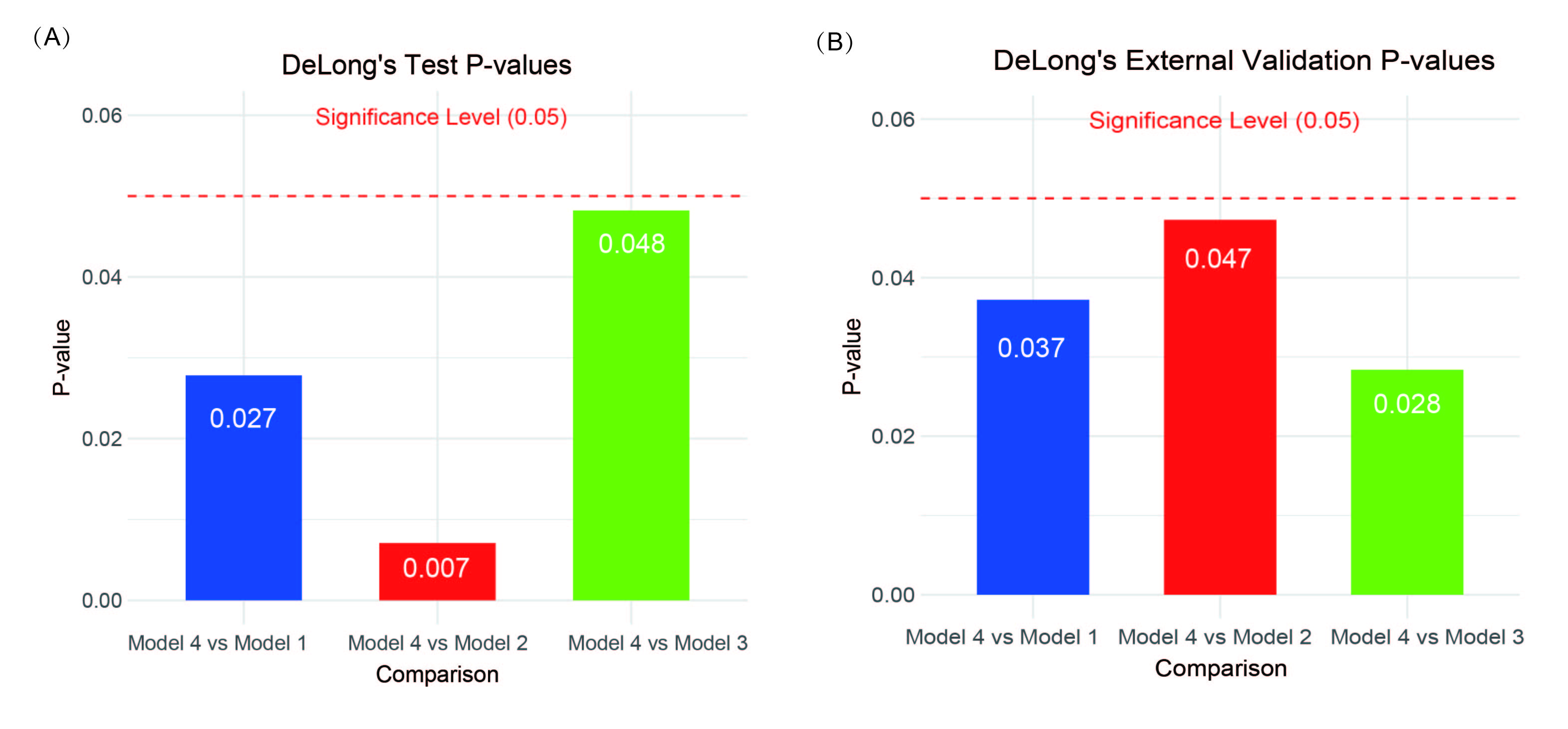

Supplement: Supplementary Figure 1 — DeLong’s test was performed to compare the AUCs between different models in the test set (A) and external validation set (B). (Model 1: Radiomics model; Model 2: Clin-US model; Model 3: Clin-Rad model; Model 4: Clin-US-Rad model) [file Image1.jpeg]

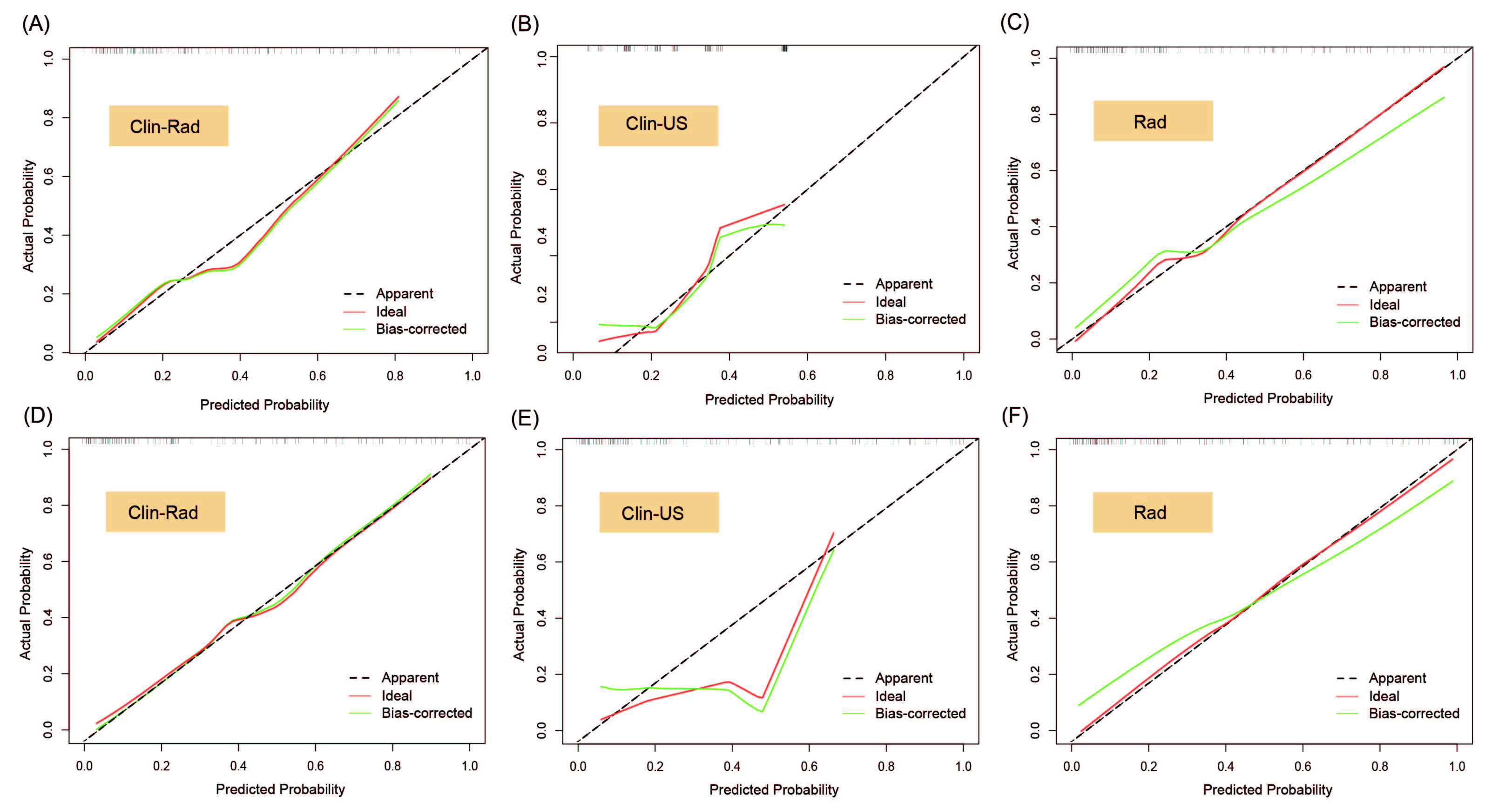

Supplement: Supplementary Figure 2 — Calibration curves of different models for predicting breast cancer recurrence risk in the test (A–C) and external validation sets (D–F). [file Image2.jpeg]

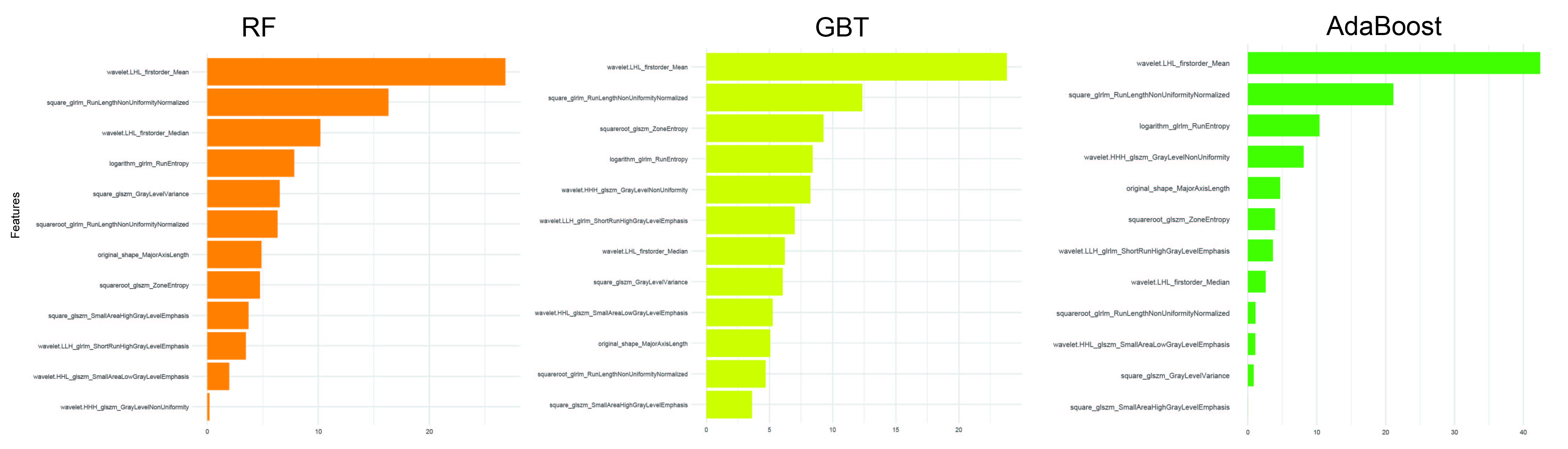

Supplement: Supplementary Figure 3 — Importance ranking of key features in radiomics weighted bar charts of RF, GBT, and AdaBoost models. [file Image3.jpeg]
